# Pathways of cellular internalisation of liposomes delivered siRNA and effects on siRNA engagement with target mRNA and silencing in cancer cells

**DOI:** 10.1038/s41598-018-22166-3

**Published:** 2018-02-28

**Authors:** Abdullah Alshehri, Anna Grabowska, Snow Stolnik

**Affiliations:** 1 Division of Molecular Therapeutics and Formulation, School of Pharmacy, University of Nottingham, Nottingham, NG7 2RD UK; 2 Cancer Biology, Division of Cancer and Stem Cells, School of Medicine, Queen’s Medical Centre, University of Nottingham, Nottingham, NG7 2RD UK

## Abstract

Design of an efficient delivery system is a generally recognised bottleneck in translation of siRNA technology into clinic. Despite research efforts, cellular processes that determine efficiency of siRNA silencing achieved by different delivery formulations remain unclear. Here, we investigated the mechanism(s) of cellular internalisation of a model siRNA-loaded liposome system in a correlation to the engagement of delivered siRNA with its target and consequent silencing by adopting siRNA molecular beacon technology. Probing of cellular internalisation pathways by a panel of pharmacological inhibitors indicated that clathrin-mediated (dynamin-dependent) endocytosis, macropinocytosis (dynamine independent), and cell membrane cholesterol dependent process(es) (clathrin and caveolea-independent) all play a role in the siRNA-liposomes internalization. The inhibition of either of these entry routes was, in general, mirrored by a reduction in the level of siRNA engagement with its target mRNA, as well as in a reduction of the target gene silencing. A dramatic increase in siRNA engagement with its target RNA was observed on disruption of endosomal membrane (by chloroquine), accompanied with an increased silencing. The work thus illustrates that employing molecular beacon siRNA technology one can start to assess the target RNA engagement – a stage between initial cellular internalization and final gene silencing of siRNA delivery systems.

## Introduction

Delivery of siRNA to the cytoplasm of target cells is a promising therapeutic approach for the treatment of a wide range of diseases^1^. However, the therapeutic potential of siRNA has not yet been realized due to the need for an appropriate delivery system^2^. The optimal siRNA delivery system should be non-toxic, protect siRNA from RNase degradation, facilitate intracellular uptake followed by escape from endosome vesicles into the cytosol, and finally encourage effective gene silencing^3^. Cationic liposomes have been reported as one of the extensively used non-viral delivery systems^4,5^ exploited in a delivery of different nucleic acids, including siRNA. In order to engineer a well-designed siRNA liposome formulation, it is important to understand the cellular internalisation and processing mechanisms of the delivery system in order to achieve efficient cellular uptake and evade the destructive or recycling cellular pathways^6^. However, despite the extensive efforts to translate siRNA-liposome technology to clinic, processes that govern interaction with cells, cellular internalisation mechanisms, and intracellular trafficking remain poorly understood^7,8^ to be exploited in rational design and engineering of efficient siRNA delivery systems.

In this study we selected to use pharmacological inhibitors to study cellular transport mechanisms, as their application is well established in the field^9^. We do acknowledge the fact that these can exert multiple cellular effects and that siRNA knockdown of endocytosis pathway-selective proteins could be advocated as an alternative^10^. However, the latter approach is less well established, has its own drawbacks^11^ and is technically impractical in the present study.

The present work exploits a relatively new siRNA molecular beacon technology, initially introduced as nucleic acid analytical probes that recognize and report the presence of specific nucleic acids in homogeneous solutions^12^, and underused in a field of siRNA delivery. siRNA molecular beacons are hairpin shaped single stranded nucleic acid probes with conjugated fluorophore and quencher that fluoresce only upon hybridization with its target mRNA molecule when the loop region hybridizes with the target, opens up and hence separates the conjugated fluorophore and the quencher. Recently application of the technology to study cellular pharmacokinetics and pharmacodynamics of siRNA intracellularly was pioneered by a seminar work in RJ Lee’s group^13^. The present study applies the technology to assess an intermittent point - the siRNA engagement with the target mRNA - in the study aiming to establish a corroboration between cellular internalisation mechanism(s), engagement in the RISC machinery, and silencing efficiency of liposomally delivered siRNA.

## Results

### Liposome formulation optimization and Cy3-Annexin V/Propidium Iodide Cytotoxicity Study

The optimization of physicochemical properties of siRNA loaded liposomes used in this study is summarized in Supplementary Information (Figs S1–S3). Empty liposomes with average particle size of ~80–100 nm were formulated by a classical ‘film hydration’ method with a range of DC-Chol to DOPE ratios (Fig. S1). Incorporation of siRNA during the liposomes fabrication, rather than subsequent addition, resulted in a good incorporation efficiency at optimized N/P ratios; for instance, the N/P ratio 3.125:1 resulted in the absence of free siRNA in the gel retardation assay (Fig. S2). An increase in the average particle size of the liposomes, with resultant sizes in the approximately 200–300 nm size range, was observed on siRNA incorporation (Fig. S3). Zeta potential values of the siRNA-liposomes were dependent on the N/P ratios used, and increased as the N/P ratio was increased; for example values in order of approximately +20 to +30 mV for N/P 3.125:1 to 12.5:1 were measured for the liposomes with DC-Chol:DOPE ratio of 1:1.

**Figure 1:**
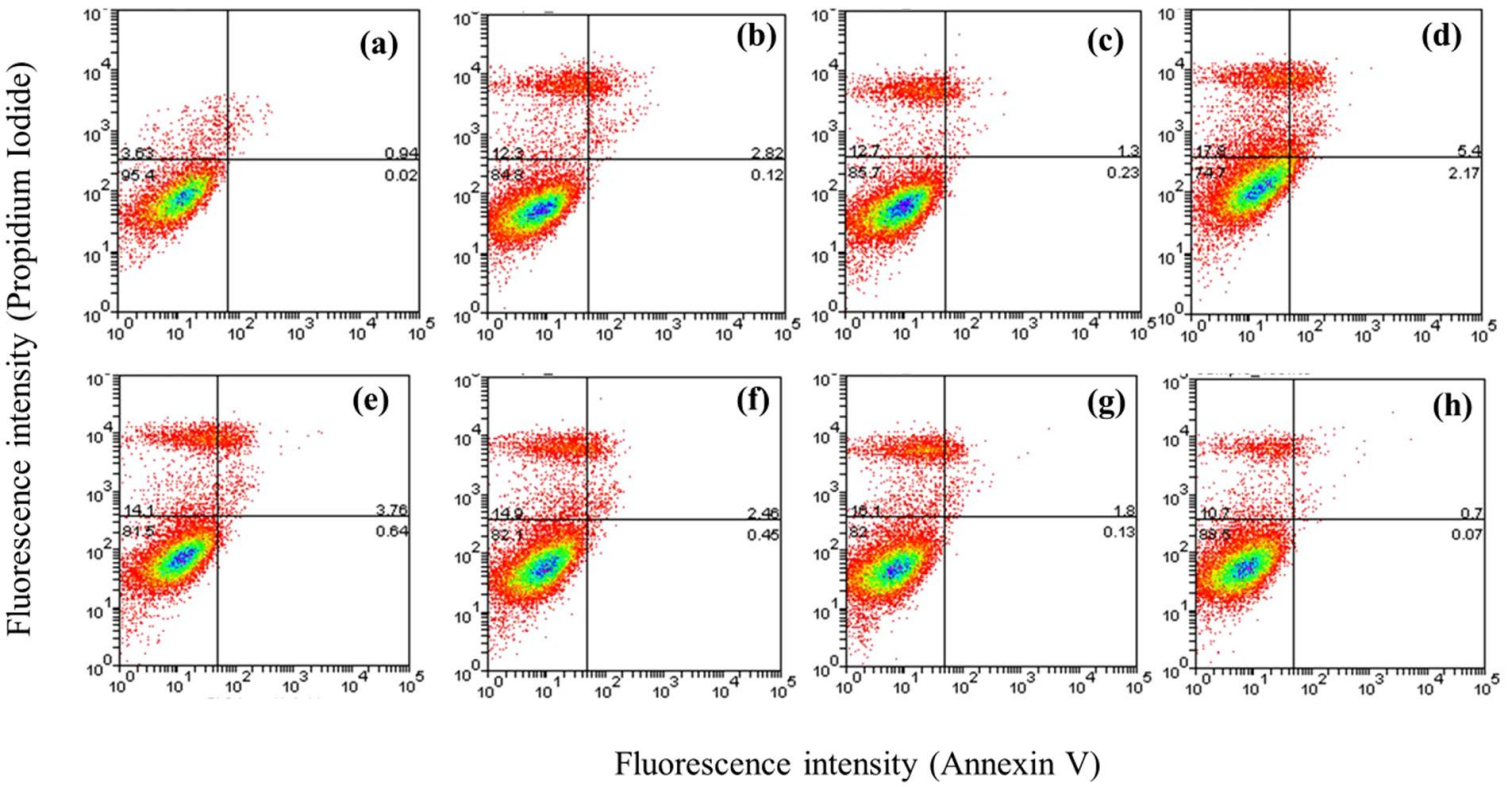
Flow cytometry dot plots of Cy3-Annexin/PI cytotoxicity test for siRNA-liposomes treatmet of A549 cells. Flow cytometry dot plots for necrosis (PI) and cell apoptosis (Cy3-Annexin) probes in A549 cells after 4 hours incubation with siRNA-liposomes at total lipid concentration of 1.0 mM, fixed N/P ratio of 3.125:1, and different DC-Chol:DOPE ratios: (**b**) 0.33:1, (**c**) 0.5:1, (**d**) 0.66:1, (**e**) 1:1, (**f**) 1.5:1, (**g**) 2:1 and (**h**) 3:1. A549 cells not treated with siRNA-liposomes used as negative control (a). The minimum 10,000 cells/sample analysed.

Toxicity of empty liposomes, as determined by MTS and PI/Annexin tests, is summarized in the Supplementary Information (Figs S4 and S5). Total lipid content applied to cells was found a crucial toxicity factor with, in general, applied concentrations above 1 mM causing a significant toxicity (EC_50_ in 24.0 to 8.7 mM range, dependent on the increase in the DC-Chol:DOPE ratio). PI/Annexin V flow cytometry dot plot profiles (Fig. S5), demonstrate increased levels of necrosis (PI staining) as the concentration of applied lipid increases above 1 mM. Cellular toxicity of ‘loaded’ siRNA-liposomes prepared at an N/P ratio of 3.125:1, different ratios of DC-Chol:DOPE and applied to the cells at 1 mM total lipid content is shown in Fig. 1. As with empty liposomes, the plots show a small, separate subpopulation of the cells that was positively stained with PI for all applied systems, rather than a whole population shift. Importantly, no significant staining with Annexin V was observed under these conditions, suggesting that no apoptosis was induced in the cells exposed to the formulations for 4 hours. As a number of endocytosis inhibitors utilised in the study acts on the cholesterol located in cellular membrane, e.g. nystatin and MßCD, LDH test was employed to confirm conditions under which the tested liposomes did not produce plasma membrane damage (Fig. S6). To optimize timing of luciferase knockdown analysis, siRNA-liposomes (at 1 µg/well siRNA, Fig. S7) were applied to A549-*luc* cells for 4 hours, removed and the assessment of luciferase activity conducted at 24, 48, 72, and 96 hours post-transfection. (No significant effects of non-targeting siRNA or effects of siRNA applied as ‘free’, unformulated solution on the luciferase activity were observed in a preliminary experiment, Fig. S7). The data (Fig. S8) indicate the highest significant decrease (p < 0.001) in luciferase activity at 48 hours post-transfection, with the recovery of luciferase activity at the later time points. All subsequent experiments were hence performed at 48 hour time point. Biophysical attributes of siRNA liposomal formulation used in all subsequent experiments are summarized in Table 1.

**Table 1:** Biophysical attributes of utilized liposomal formulation.

Attribute	Value
Lipid composition (mol ratio)	1:1 molar ratio of DC-Chol: DOPE
Average hydrodynamic diameter ± SD (nm)	207.9 ± 26.8
Zeta potential ± SD (in 10 mM PBS) (mV)	+28.8 ± 1.5
EC_50_ by MTS assay (mM)	8.69
N/P ratio of siRNA loaded formulation	3.125:1

### Mechanisms of Cellular Internalisation of ^Cy3^siRNA-Liposomes

**Figure 2:**
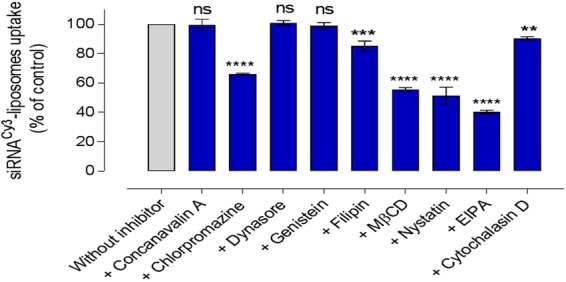
The effect of a panel of pharmacological inhibitors on the internalisation of ^cy3^siRNA-liposomes in A549 cells. Cellular uptake of ^cy3^siRNA-liposomes in absence (‘without inhibitor’ control) and presence of inhibitors, assessed by flow cytometry (MFI). Data represent the mean ± SD (N = 2, n = 4). **, *** and **** indicate a significant difference between the results (p < 0.01, p < 0.001 and p < 0.0001, respectively, and *ns* indicates the difference is non-statistically significant (p > 0.05) compared to the control without inhibitor treatment.

**Figure 3:**
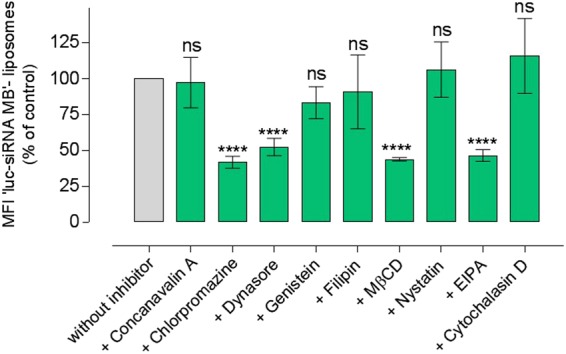
The effect of pharmacological inhibitors on the engagement of liposomes delivered *luc*-siRNA-molecular beacon (MB) with target mRNA in A549-*luc* cells. *Luc*-siRNA-liposomes prepared at an N/P ratio 3.125:1, DC-Chol:DOPE ratio of 1:1, applied at 1 μg of *luc*-siRNA *per* well and at 1 mM total lipid content. Fluorescence from flow cytometry experiments expressed relative to the control (cells without inhibitors representing 100%); data represent the mean ± SD (N = 2, n = 4), **** indicate a significant difference between the results (p < 0.0001) and *ns* indicates the difference is a non-statistically significant (p > 0.05) compared to the control. Confocal microscopy micrographs show cells treated with ‘*luc*-siRNA based Molecular Beacon’ (MB) liposomes in A549-*luc* cells in the absence or presence of certain inhibitors. Engaged ‘*Luc*-siRNA Molecular Beacon’ siRNA appears green, whereas nuclei appear blue, staining with DRAQ5.

**Figure 4:**
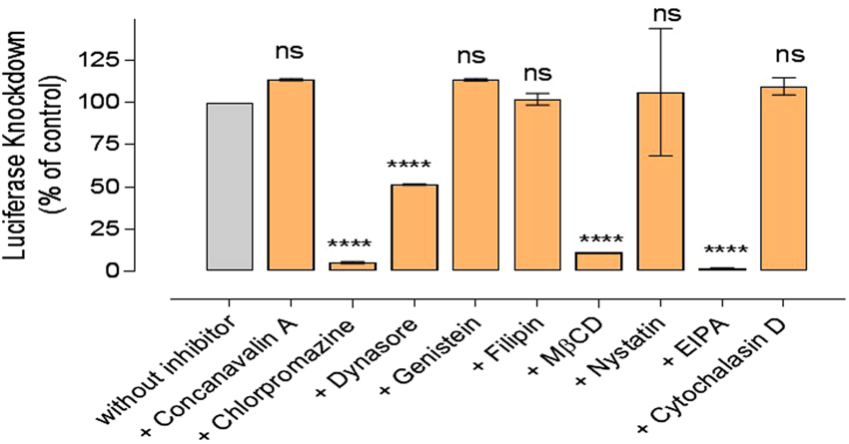
The effect of pharmacological inhibitors on the luciferase knockdown by *luc*-siRNA-liposomes in A549-*luc* cells. *Luc*-siRNA-liposomes prepared at an N/P ratio 3.125:1, DC-Chol:DOPE ratio of 1:1, applied at 1 μg of *luc*-siRNA *per* well and 1 mM total lipid content. The *luc*iferase activity assessed after 48 hours. Luciferase knockdown relative to the control (cells without inhibitors as 100%), data represents the mean ± SD (N = 2, n = 4), **** indicate a significant difference between the results (p < 0.0001) and *ns* indicates the difference is a non-statistically significant (p > 0.05) compared to the control.

We used pharmacological inhibitors of endocytosis to assess cellular processing of siRNA-liposomes. The effect of endocytosis inhibition on the cellular uptake of ^cy3^siRNA-liposomes in A549 cells was examined by flow cytometry following the application of concanavalin A (100 μg/ml), chlorpromazine (20 μg/ml), dynasore (20 μg/ml), genistein (15 μg/ml), filipin (20 μg/ml), MßCD (300 μg/ml), nystatin (20 μg/ml), EIPA (0.5 μg/ml), and cytochalasin D (2 μg/ml). Concertation optimization of used inhibitors is shown in Supplementary Information (Fig. S9) and, when available, ligands known to selectively use a specific entry pathway (transferrin for clathrin and choleratoxin B for caveolae mediated endocytosis) were used as positive controls to confirm the effect of applied inhibitors (Fig. S10). The data show that the inhibitory effect is increased with a corresponding increase in the concentration of inhibitors tested. Regarding inhibition of macropinocytosis by EIPA and cytochalasin D, as dextran may not to be a solely specific ligand for this internalisation pathway^14^, we tested these inhibitors at three different sub-EC_50_ concentrations to eliminate possible artefacts of a singular concentration, as shown in Figure S11. The levels cell viability, determined as cell metabolic activity by MTS assay, at the above listed concentrations of endocytosis inhibitors applied are summarized in Supplementary Information (Tbl. S1). Tested at corresponding experimental conditions, these data allow comparisons of treated with control sets of data in Figs 2–4. Cellular internalization of liposomes was, in addition to flow cytometry, visualized by confocal microscopy (e.g. Fig. S12). Experimental data are summarized in Fig. 2.

Considering first an inhibition of clathrin-mediated pathway, concanavalin A - an inhibitor that interferes with the signalling of transmembrane G protein-coupled receptors located on the surface of cells, and in that way prevents the assembly of clathrin coated pits and impairs their movement through the cell membrane - had no significant effect (p > 0.05) on the internalisation of ^Cy3^siRNA-liposomes by A549 cells (Fig. 2). In contrast, chlorpromazine inhibition of clathrin pathway, which acts by a different mechanism of preventing formation of clathrin-coated pits at the plasma membrane, demonstrated a significant effect (p < 0.0001) on the internalization, reducing it to approximately 66% of that in untreated cells.

The inhibition of dynamin by dynasore, an inhibitor with the ability to block the GTPase activity of dynamin and hence detachment from the membrane in dynamin-dependent endocytosis in cells, exhibited no significant effects (p > 0.05) on the internalisation of ^cy3^siRNA-liposomes. Genistein, as tyrosine kinase and caveolae pathway inhibitor, did not produce a statistically significant effect on the internalisation of ^cy3^siRNA-liposomes. However, inhibition of caveolae-mediated endocytosis by filipin showed a moderate, statistically significant reduction in the internalisation of ^cy3^siRNA-liposomes (p = 0.0001). MßCD inhibitor, which depletes cell membrane associated cholesterol, reduced the internalisation of ^cy3^siRNA-liposomes significantly (p < 0.0001). Another inhibitor that acts on membrane cholesterol, nystatin, also demonstrated a significantly reduced liposomes internalisation (p < 0.0001), 51% of untreated cells. EIPA inhibits Na+/H+ exchange, consequently lowering the sub-membranous pH of the cell cytoplasm, and is normally used to investigate the role of macropinocytosis pathway. Results show that EIPA was able to significantly reduce the internalisation of ^cy3^siRNA-liposomes (p < 0.0001). Moreover, the inhibition of endocytosis by cytochalasin D, another macropinocytosis inhibitor, showed a moderate, but statistically significant reduction (p < 0.01).

### Effect of endocytosis inhibitors on engagement of liposomes delivered *luc*-siRNA-molecular beacon with target mRNA

Flow cytometry data summarized in Fig. 3 demonstrate effects of different pharmacological inhibitors on the *luc*-siRNA-molecular beacon engagement (in terms of the inhibitor’s effect on measured engagement, i.e. cell associated fluorescence, relative to untreated control) with target luciferase mRNA in A594-*luc* cells following application of tested liposomal formulation. On the whole, the levels of siRNA engagement with its target mRNA follow the trend obtained for inhibition of liposomes internalization in Fig. 2, with the exceptions of dynasore and nystatin. For example, a pronounced inhibition of the internalization was seen for chlorpromazine, MβCD and EIPA treatments (Fig. 2), and the same inhibitors produced a statistically highly significant reduction in the molecular beacon siRNA engagement (statistical significance as indicated in Fig. 3), while concavalin A, genistein and cytochalasin did not produce a statistically significant reductions in either cellular internalization or siRNA engagement with target mRNA (statistical significance as indicated). Confocal micrographs in Fig. 3 visualize the presence of engaged, hence green *luc*-siRNA based molecular beacon in A549-*luc* cells for some tested systems. The fluorescence broadly appears to be localised in the perinuclear region of the cells.

### Effect of endocytosis inhibitors on siRNA-liposomes silencing

Figure 4 presents the effect of endocytosis inhibition on silencing of luciferase protein expression in A549-*luc* cells. The general data profile appears to follow trends in Figs 2 and 3; the main feature being the effect of dynasore; it statistically significantly (p < 0.0001) reduced molecular beacon siRNA engagement with its target mRNA (Fig. 3) and produced a significant luciferase silencing effect (p < 0.0001), despite no significant effect (p > 0.05) on the cellular uptake (Fig. 2). A highly significant reduction of the silencing effect was measured for chlorpromazine, MßCD and EIPA treatments (statistical significance as indicated in Fig. 4), whilst concavalin A, genistein, nystatin and cytochalasin D did not produce a significant reduction in the silencing effect (all p > 0.5).

**Figure 5:**
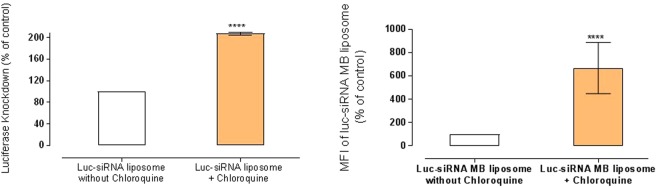
The effect of endosomolytic agent, chloroquine, on the engagement of liposomes delivered *luc*-siRNA-molecular beacon (MB) with target mRNA luciferase and luciferase knockdown in A549-*luc* cells. *luc*-siRNA-liposomes prepared at an N/P ratio of 3.125:1, a DC-Chol: DOPE ratio of 1:1. Liposomes applied in the presence of chloroquine (100 μg/ml). Data presented relative to appropriate control, cells without chloroquine taken as 100%. Data represent the mean ± SD (N = 2, n = 4), **** indicate a significant difference between the results (p < 0.0001) and *ns* indicates the difference is a non-statistically significant (p > 0.05) compared to the control.

Figure 5 shows the effect of chloroquine (100 μg/ml) on the luciferase silencing by *luc*-siRNA-liposomes in A549-*luc* cells. The data clearly demonstrate that chloroquine treatment statistically significantly (p < 0.0001) increases both the target RNA engagement and luciferase knockdown, relative to the cells not treated with chloroquine; the results show approximately 7-fold increases in fluorescence signal of the ‘*luc*-siRNA based MB’ in comparison to control and approximately 2-fold increase in the silencing.

## Discussion

Despite extensive research in the design of formulations to deliver siRNA, the cellular internalisation pathways of different siRNA formulations, including liposomes, and engagement of delivered siRNA with its target mRNA, have not been extensively studied to provide a mechanistic insight on cellular processing and, consequently, define requirements for, and functionalities needed of, an optimized siRNA delivery formulation^15^. A substantial body of literature concludes that siRNA-liposomes enter cells *via* endocytosis pathways^7,16,17^. More specifically, it has been proposed that cationic liposomes (containing helper lipid as in the present study) are internalised by the clathrin-mediated pathway, leading to a relatively successful gene knockdown. The success is generally attributed to the interactions of charged lipids with the anionic components of endosomal membrane, and the effects of helper lipid on the cellular membranes structure, leading to the material escape from endosomal vesicles^18^. It has also been suggested that the caveolae-mediated pathway plays an essential role in a successful gene delivery, due to avoiding the delivery systems’ ‘entrapment’ in endosomes and lysosomes^19^. In either cases, further cellular processes leading to siRNA silencing remain to be deciphered. In that context, involvement of the intracellularly present (delivered) siRNA with its target mRNA is scarcely studied to bridge delivery (cellular internalization) stage with the outcome - siRNA silencing.

Here we investigated the internalisation pathways of siRNA-liposomes in a conjunction with assessments of ‘downstream’ processes: siRNA engagement with its target mRNA in the cell cytosol and eventual gene silencing effect. We applied highly sensitive and sequence specific siRNA molecular beacon technology, used in molecular biology field for, for instance, gene expression analysis, gene copy determination, sequence detection in qPCR, and recently pioneered to assessment of intracellular siRNA engagement with its target mRNA^13^. We sought to investigate if the application of this technology would provide further insight into understanding of intercellular processing of delivered siRNA.

Initially we made a considerable effort to fabricate a ‘model’ siRNA loaded liposomes with ‘optimized’ biophysical properties (Table 1); particle size appropriate for endocytosis, i.e. diameter in ~200 nm range, and surface charge of approximately +20 to +30 mV - typical features of siRNA delivery liposomal systems - and that their application to cells in culture, under optimized conditions, did not induce evident toxic effects or membrane damage. Moreover, a panel of inhibitors was selected and a certain endocytosis pathways probed *via* different inhibition mechanisms, as described in the results section.

In essence, when data presented in Figs 2, 3 and 4 are taken together a clear corroboration appears between trends for cellular uptake, engagement with target mRNA, and siRNA induced luciferase silencing as cells are treated with different pathways inhibitors; nystatin and dynasore treatments are exceptions, as will be discussed later.

In more details, probing clathrin-mediated endocytosis by applying chlorpromazine reduced siRNA-liposomes uptake to 66% of the control, target mRNA engagement to 41%, and silencing to 5% of the luciferase knockdown seen in the absence of the inhibitor, whilst concavalin A did not produce any significant effect. In our previous work, concanavalin A (at 100 μg/ml) reduced internalisation of siRNA-polyplexes with modified chitosan polymers by A549 cells to approximately 50% of untreated control^20^, while chlorpromazine was reported to reduce the internalisation DOTAP lipoplexes, also in A549 cells^21^. However, to the best of our knowledge, there is no a report on the use of both of these inhibitors of clathrin-mediated endocytosis in one study to make comparisons and possibly explain the apparently contradictory effects. One could hypothesise that the different outcomes observed could possibly be due to different mechanisms these inhibitors use to affect the clathrin-mediated pathway^22,23^, but a further investigation would be needed to decipher the reason(s).

Treatment with dynasore, to inhibit dynamin-dependent entry routes, showed that, despite no significant effect on the liposomes cellular uptake, the internalized siRNA engagement with its target mRNA was reduced to ~52%, and silencing to ~51% of the respective untreated controls. Dynasore is a pharmacological inhibitor of dynamin, a GTP-dependent enzyme that forms helical ‘collar’ around the neck of newly formed cell membrane invagination inducing a scission of endocytic vesicles from the cell membrane; it plays an essential role in both caveolin and clathrin-coated pathways and some lipid raft-mediated processes^24^. The data presented here could indicate that, although internalization of the liposomes occurred in the presence of dynasore, a significant reduction in the molecular beacon siRNA engagement with target mRNA occured due to inhibition of endocytic vesicles scission from the cell plasma membrane which, in turn, prevented their further intracellular trafficking and hence downstream effects of the internalized siRNA. It should be noted however that other cellular entry pathways not dependent on dynamin would not be inhibited by dynasore, and their possible involvement (as discussed later) would result in the liposomal uptake and ‘downstream’ effects (siRNA engagement with mRNA and silencing) observed. Taken together with the data on chlorpromazine inhibition, as well as chloroquine treatment (Fig. 5), this would point to a conclusion that dynamine dependent i.e. a vesicular processes (clathrin and/or caveole-mediated) could play an important role in internalization of the siRNA-liposomes and consequent cellular effects. One should however appreciate that chloroquine treatment has been reported to inhibit clathrin-dependent endocytosis and that the effects observed in Fig. 5 can only be taken as an overall balance of its influences^25^.

Probing of caveolae-mediated endocytosis however, by applying ‘classical’ inhibitors, genistein (tyrosine kinase inhibitor) and filipin (binds to membrane cholesterol), revealed that this pathway does not play a significant role in the siRNA-liposomes internalization, and does not affect siRNA engagement with target mRNA or luciferase silencing. This would hence point to the major role of clathrin-mediated pathway. Involvement of clathrin pathway in the cellular uptake of positively charged nanoparticles, as is the case with liposomes used in the present study (Table 1), would be in line with a body of literature concluding that these systems are internalised through clathrin-mediated endocytosis^26,27^, although some studies advocate the entry *via* caveolae- and clathrin-independent endocytosis^28^, macropinocytosis^29^ or multiple pathways including caveolae-mediated endocytosis^30^.

Methyl-β-cyclodextrin, MβCD, is a cholesterol-solubilising agent that ‘extracts’ cholesterol from the plasma membrane by forming soluble cholesterol inclusion complexes^31^, thus changing the structure of cholesterol rich domains within the membrane^32^; it reportedly disrupts a number of processes involving cholesterol including lipid rafts, macropinocytosis^33^ as well as caveolae-mediated uptake^34^. With this broad spectrum of action in mind, its significant reduction of the liposomes internalization (~55% of untreated control), translated into a significant reduction of both siRNA engagement (~43%) and silencing effect (~11% of untreated control), indicates an important role of cholesterol and the cell membrane structure on the internalization of the tested liposomes. Similarly, cellular uptake of liposomes was significantly reduced in the presence of MβCD in two cell lines, COS-7 and HUVEC in another study^35^. As indicated above, cholesterol involvement is reported in a classical caveolae-dependent endocytosis, but also in macropinocytosis and in much less understood clathrin and caveolae-independent processes, making an unequivocal conclusion difficult. However, treatments with other two compounds, filipin and nystatin, that act based on their *binding to* the membrane cholesterol, rather than cholesterol *depletion from* the membrane, resulted in a moderate or significant reduction of cellular uptake (to ~85% for filipin and to ~51% for nystatin), but with no measureable effects on either siRNA engagement or luciferase silencing. Similarly another study on DNA/DOTAP lipoplexes demonstrated that filipin had no significant effect on their internalisation^36^. These two inhibitors are normally considered to principally influence caveolae-mediated internalization, and the results on this work would be in line with this proposition^37,38^. In addition, effects of the latter two inhibitors also eliminate influences of a possible disruptive effect of MβCD on the liposomes used in the study, and significant effects on liposomes internalization. It would require further mechanistic study to ascertain possible complexation of CD-cholesterol with MβCD, and if interactions of siRNA with CD-cholesterol diminish such complexation.

Considering other endocytic pathways, EIPA (5-(N-ethyl-N-isopropyl)-amiloride) is generally accepted to inhibit macropinocytosis by inhibiting the Na+/H− exchange located in membranes in a dynamin-independent way. The significant reduction of liposomes uptake to 40%, siRNA engagement to 46%, and luciferase silencing to 2% of relevant untreated controls all illustrate a major role of this internalization pathway on the siRNA delivery and knockdown of tested liposomal formulation. The prominent role of macropinocytosis seen in this study would be in line with a recent report where a down-regulation of macropinocytosis components (Cdc42 and Rac1) in HeLa cells decreased the internalization of siRNA-loaded cationic lipid nanoparticles by ~80%, whereas inhibition caveolae-dependent endocytosis and clathrin pathway (contrary to the present study) had little impact on cell entry^39^. (The possible effects of cell types used in the studies should be noticed to account for observed differences in the clathrin inhibition^20,39,40^). Similarly, it has been reported that the internalisation of liposomes was reduced significantly by EIPA in murine bladder tumour cells (MBT-2)^41^, whilst it did not have an effect on the uptake of liposomes in Zebrafish ZFL cells^42^.

It should be noted that, on the contrary to EIPA, cytochalasin D, another recognized macropinocytosis inhibitor - an actin filament depolymerising agent that inhibits macropinocytosis by binding to the positive termini of actin filaments and preventing the elongation of microfilament^43^ - showed no significant effect on either of tested processes. At this stage, we cannot propose a clear explanation. Possible discussion may consider that, in addition to a different mechanism of action by cytochalasin D, we observed that cells treated with this inhibitor presented a somewhat altered nuclear morphology (Fig. S13) even at a relatively low concentration used (as *per* Fig. S9); similarly, changes in cell morphology were recently reported for cytochlasin D treated A549 cells^44^.

Observed engagement of liposomes delivered siRNA with target mRNA indirectly confirms that following internalization (a population of) internalized liposomes disassembled and/or released ‘free’ siRNA. This may indicate that liposomes cause perturbations of the endocytic vesicles membrane, and a consequent siRNA ‘release’, that may be helped by the presence of ‘helper lipid’ DOPE in the liposomes formulation utilised^45,46^. The role of DOPE however does not appear to be the only, and/or the most efficient process as the cells treatment with an endosomolytic agent, chloroquine (Fig. 5) resulted in a ~7-fold increase in siRNA engagement with the target mRNA, and a ~2-fold increase in the silencing effect.

Our data point to an inefficient escape from endocytic vesicles, also previously demonstrated for lipid nanoparticles mediated siRNA delivery whereby, by directly detecting colloidal-gold particles conjugated to siRNAs, authors estimated that escape of siRNAs from endosomes into the cytosol occurs at low efficiency, at a level of 1–2%^47^.

The pronounced effect of chloroquine in our work may not be unexpected, based on the above discussion, assuming that clathrin-mediated internalization and macropinocytosis eventually associate with the lysosomes^45,48^ however some reports conclude that contents in the macropinosomes may not merge with late lysosomes^49,50^. Another point to consider is from work by Wittrup *et al*.^51^ who demonstrated that siRNA release into cell cytosol occurs during narrow ‘window of opportunity’ from maturing endosomes, and that no release occurs from late endosomes or lysosomes. They postulated that an initiated autophagy process, due to endosomal damage, may be a potential reason for the latter observation but, by inhibiting autophagy, did not manage to enhance siRNA release and silencing, hence concluding that there are other ‘actors’ in the play.

Nevertheless, the chloroquine treatment in the present work points to the importance of an efficient endosomal escape in the design of siRNA delivery systems internalized *via* clathrin-dependent pathway and, possibly, macropinocytosis. The data also demonstrate that measuring the molecular beacon florescence was able to assess a level of intracellularly ‘engaged’ siRNA with the target mRNA following the endosomolytic disruption and hence can be used as a non-invasive assay to assess the efficacy of endosomolytic functionality of siRNA delivery systems.

## Conclusions

In summary, here we present our work into following and connecting stages of therapeutic siRNA delivery from the initial pathways of cell entry, to engagement with its target RNA, and to the consequent siRNA silencing effect. A clear example of monitoring an increased siRNA engagement with target RNA on enhanced lysosomal escape (on chloroquine treatment) illustrates an application of the siRNA-molecular beacon technology in drug delivery field as a non-destructive method to study siRNA intercellularly. The work is underway to, from essentially qualitative information at this stage, obtain quantitative information on siRNA ‘cellular pharmacokinetics’ following application of different siRNA delivery systems and hence provide comparisons and inform design specifications.

## Methods

### Preparation of siRNA-Liposomes

The formulation of siRNA-liposomes was prepared using the thin-film hydration method with different molar ratio of DC-Chol (cationic lipid) to DOPE (neutral lipid) and with N/P ratio (cationic lipid/siRNA) of 3.125:1. The formulation was passed through an extruder equipped with 0.2-μm polycarbonate membrane filters for 21 times to produce unilamellar liposomes and to reduce the particle size of the formulation.

### Annexin V-Propidium Iodide Assay

Annexin V/PI assay was performed on the A549 cells to differentiate between the apoptotic, necrotic and live cells. A549 cells were seeded onto a 24-well plate at a density of 5 × 10^4^ cells per well and incubated overnight. The cells were then incubated with liposomes samples for 4 hrs, while those incubated with normal media (DMEM) were used as negative controls. Annexin V/PI assay was conducted according to the manufacturer’s instructions. The cells were harvested and assessed by flow cytometry a Beckman Coulter MoFlo (minimum 10,000 cells/sample), and data was analysed using Weasel Software Version 3.0.2 (The Walter and Eliza Hall Institute of Medical Research, Melbourne Australia).

### Assessment of siRNA-Liposomes Cellular Uptake and Luciferase Protein Silencing

A549 and A549-*luc* cells were seeded onto 24-well plates at a density of 5 × 10^4^ cells per well and cultured overnight in 0.5 mL of DMEM. Cells were then washed with PBS and the samples of siRNA-liposome formulations containing siRNA were added to the cells in OptiMEM® serum-reduced media and incubated for 4 hrs. Cellular uptake was assessed after 4 hrs using flow cytometry (minimum 10,000 events, Beckman Coulter MoFlo). The assessment of *luc*iferase activity was performed after 24, 48, 72, and 96 hrs using the *luc*iferase assay system (Promega) according to the manufacturer’s protocol.

### Study Mechanism of endocytosis of siRNA-Liposomes

To study the endocytosis mechanism of siRNA-liposomes, different endocytosis inhibitors were applied to the cells. The mechanism of endocytosis was studied by the application of inhibitors 30 min before treating the cells with siRNA-liposomes or ‘*luc*-siRNA’ liposomes samples. The inhibition was evaluated using flow cytometry, *luc*iferase assay, and confocal microscopy.

### Confocal Microscopy

A549 and A549-*luc* cells were cultured overnight in an 8-well chamber slide at a density of 3 × 10^4^ cells per chamber. Cells were then treated with cy3-siRNA liposomes (in A549 cells) or 6-FAM ‘*luc*-siRNA based Molecular Beacon’ liposomes (in A549-*luc* cells) for 4 hrs. Cells were then washed gently with PBS and then fixed with 4% paraformaldehyde for 10 min at room temperature. Washing the cells with PBS was repeated before the cells were incubated for 10 min at room temperature with DRAQ5 (1:5000) to stain the nuclei. Finally, cells were washed once again with PBS and one drop of mounting media was added. Slides were stored at 4 °C until viewed using a Zeiss LSM510 confocal microscope.

### Statistical Analysis

All data are displayed as mean ± standard deviation with three repeats. Student’s t-tests were performed for comparisons of two group means, whilst one way analysis of variance (ANOVA) followed by Bonferroni post-hoc test was applied for comparison of three or more group means. P value of < 0.05 was considered statistically significant. ****, ***, ** and * display p < 0.0001, p < 0.001, p < 0.01 and p < 0.05, respectively, whereas ‘ns’ indicates non-significant.

## Electronic supplementary material

supplementary information
