# Publisher Correction: Pathways of cellular internalisation of liposomes delivered siRNA and effects on siRNA engagement with target mRNA and silencing in cancer cells

**DOI:** 10.1038/s41598-018-24860-8

**Published:** 2018-04-25

**Authors:** Abdullah Alshehri, Anna Grabowska, Snow Stolnik

**Affiliations:** 1 Division of Molecular Therapeutics and Formulation, School of Pharmacy, University of Nottingham, Nottingham, NG7 2RD UK; 2 Cancer Biology, Division of Cancer and Stem Cells, School of Medicine Queen’s Medical Centre, University of Nottingham, Nottingham, NG7 2RD UK

Correction to: *Scientific Reports* 10.1038/s41598-018-22166-3, published online 28 February 2018

This Article contains an error in Figure 3, in which the confocal microscopy micrograph images are missing. The correct Figure 3 appears below as Figure 1.

**Figure 1:**
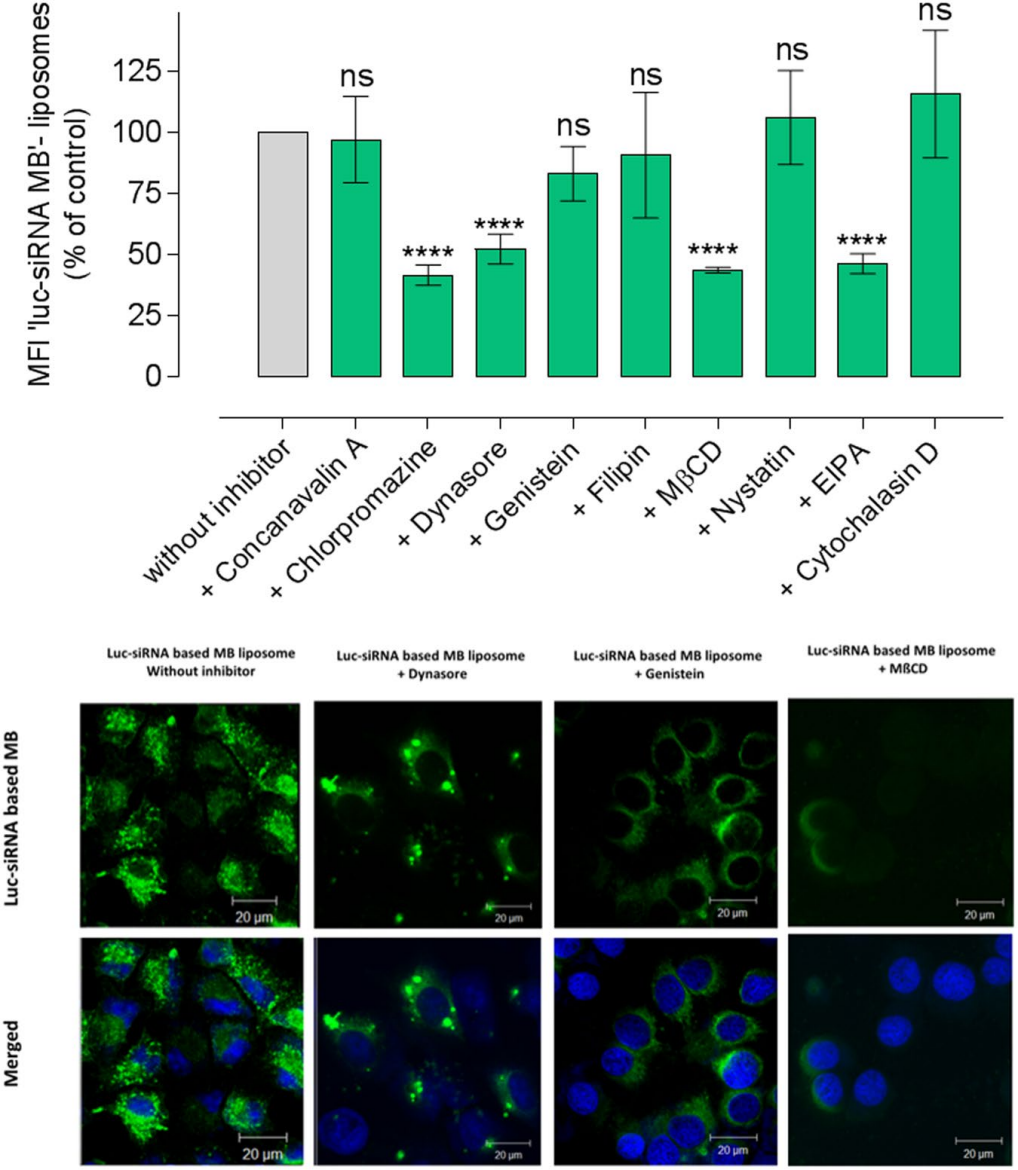
The effect of pharmacological inhibitors on the engagement of liposomes delivered *luc*-siRNA-molecular beacon (MB) with target mRNA in A549-*luc* cells. *Luc*-siRNA-liposomes prepared at an N/P ratio 3.125:1, DC-Chol:DOPE ratio of 1:1, applied at 1 μg of *luc*-siRNA *per* well and at 1 mM total lipid content. Fluorescence from flow cytometry experiments expressed relative to the control (cells without inhibitors representing 100%); data represent the mean ± SD (N = 2, n = 4), **** indicate a significant difference between the results (p < 0.0001) and *ns* indicates the difference is a non-statistically significant (p > 0.05) compared to the control. Confocal microscopy micrographs show cells treated with ‘*luc*-siRNA based Molecular Beacon’ (MB) liposomes in A549-*luc* cells in the absence or presence of certain inhibitors. Engaged ‘*Luc*-siRNA Molecular Beacon’ siRNA appears green, whereas nuclei appear blue, staining with DRAQ5.

