# Supplementary material for: Pathways of cellular internalisation of liposomes delivered siRNA and effects on siRNA engagement with target mRNA and silencing in cancer cells
Source: Sci Rep. 2018 Feb 28;8:3748. doi: 10.1038/s41598-018-22166-3 (PMC5830644; doi:10.1038/s41598-018-22166-3)
Supplement: Supplementary file 1 — supplementary information [file 41598_2018_22166_MOESM1_ESM.docx]

**Pathways of cellular internalisation of liposomes delivered siRNA and effects on siRNA engagement with target mRNA and silencing in cancer cells**

**Abdullah Alshehri^1^, Anna Grabowska^2^, and Snow Stolnik^1,*^**

*^1^ Division of Molecular Therapeutics and Formulation, School of Pharmacy, Boots Science Building, University of Nottingham,* *Nottingham NG7 2RD, U.K.*

*^2^ Cancer Biology, Division of Cancer and Stem Cells, School of Medicine, Queen's Medical Centre, University of Nottingham, Nottingham NG7 2RD, U.K.*

*snow.stolnik@nottingham.ac.uk

**Corresponding author:** Dr Snow Stolnik

Associate Professor in Advanced Drug Delivery, Faculty of Science, Division of Molecular Therapeutics and Formulation, School of Pharmacy, Boots Science Building, University of Nottingham, Nottingham NG7 2RD, U.K

**Supplementary Figure S1. Hydrodynamic diameter (nm) and zeta potential (mV) for empty liposomes prepared at different compositions and N/P ratios.** Empty liposomes were prepared with different DC-Chol:DOPE ratios at 1.0 mM total lipid concentration. Mean hydrodynamic diameter (n = 10 ± SD) of the formulations were measured by Dynamic Light Scattering (DLS) using a Viscotek DLS 802 system. Zeta potential measurements (n = 3 ± SD) were carried out at 25.0°C in 10 mM PBS solution (pH 7.4) using Zetasizer Nano Series (Malvern Instruments, UK).


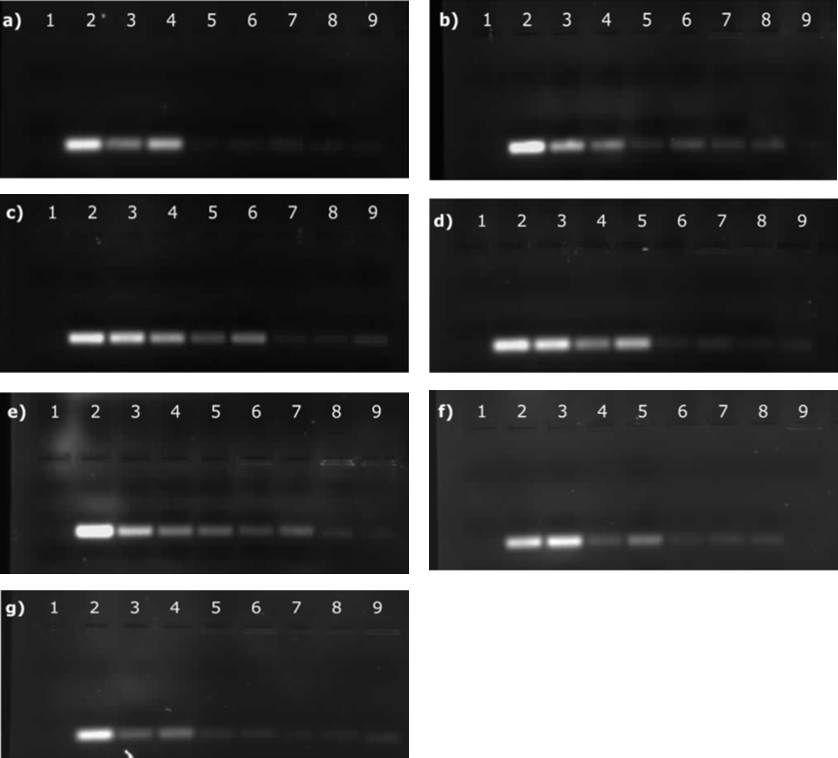


**Suplementary Figure S2.** **Encapsulation of siRNA into liposomes, as assessed by gel retardation assay.** Cationic liposomes were prepared with different DC-Chol:DOPE composition of (a) 0.33:1, (b) 0.5:1, (c) 0.66:1, (d) 1:1, (e) 1.5:1, (f) 2:1 and (g) 3:1. In each gel, liposome–siRNA formulations were tested at various N/P ratios: 0.78:1, 1.56:1, 3.125:1, 6.25:1, 12.5:1, 25:1, and 50:1 (lanes 3, 4, 5, 6, 7, 8 and 9, respectively). Lanes 1 and 2 represent the controls and contain empty liposomes and naked siRNA (0.4 μg), respectively. Individual, full-length (not cropped) gels, with white spaces in the figure indicating delineation of individual gels, are shown.

**Supplementary Figure S3.** **Hydrodynamic diameter (nm) and zeta potential (mV) for siRNA-liposomes prepared at different compositions and N/P ratios.** siRNA-liposomes prepared with different DC-Chol:DOPE compositions and at different N/P ratios (0.78:1, 1.56:1, 3.125:1, 6.25:1, 12.5:1, 25:1, and 50:1. Mean hydrodynamic diameter (n = 10 ± SD) of the formulations determined Dynamic Light Scattering (DLS) using a Viscotek DLS 802 system. Zeta potential measurements (n = 3 ± SD) were carried out in 10 mM PBS solution (pH 7.4) using Zetasizer Nano Series (Malvern Instruments, UK).

**Supplementary Figure S4. Dose-response profiles for relative cell viability following application of empty liposomes in A549 cells.** Data from the MTS assay; expressed as relative cell viability and presented as the mean ± SD (N=3, n=6). Different concentrations of cationic liposomes (1.0, 5.0, 10.0, 25.0, and 50.0 mM) were used at different ratios of a DC-Chol:DOPE and incubated with cells for 4 hrs: a) 0.33:1, b) 0.5:1, c) 0.66:1, d) 1:1, e) 1.5:1, f) 2:1 and g) 3:1. Dose-response curves were generated using GraphPad Prism (v6).

**
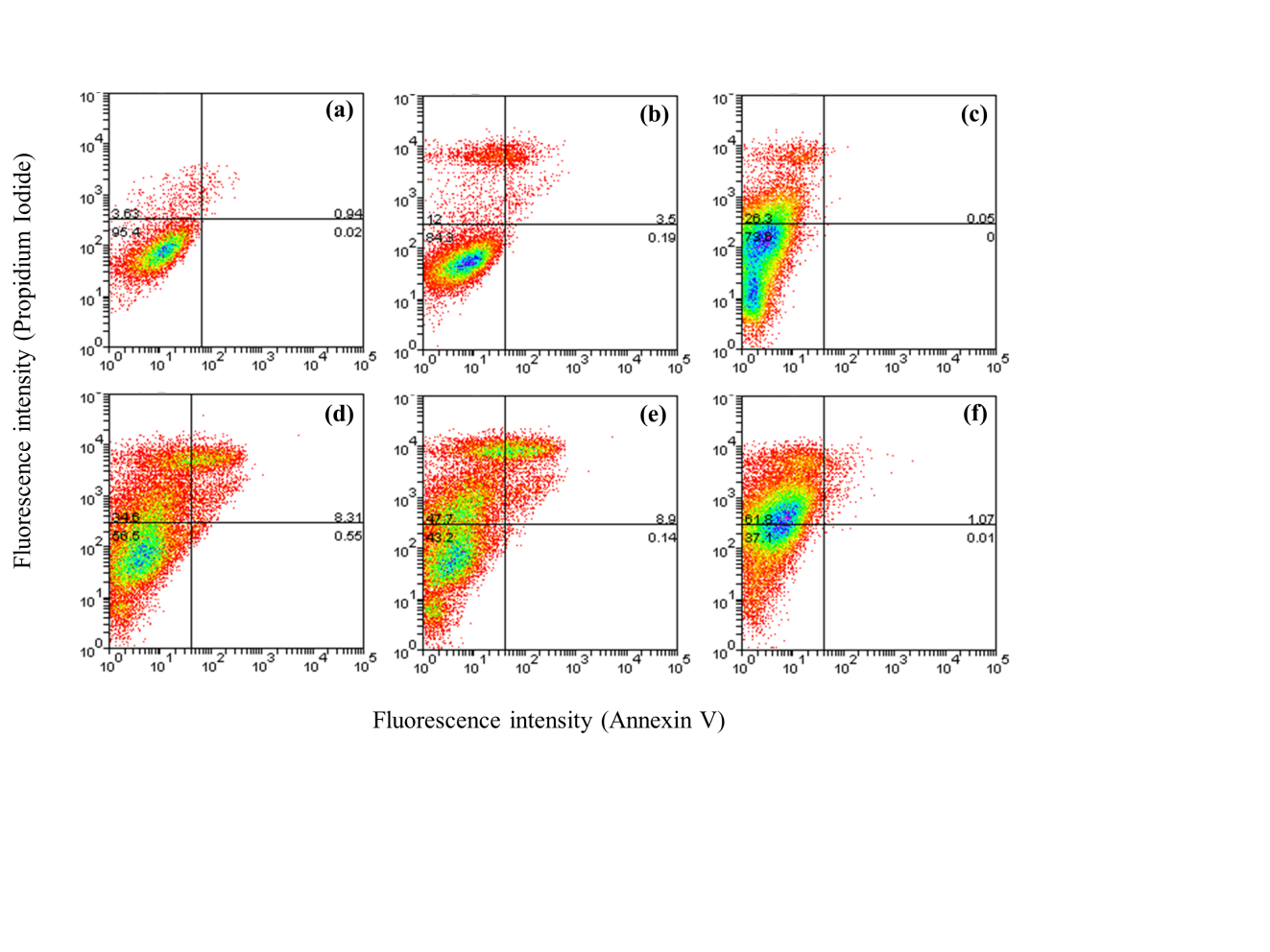
**

Supplementary Figure S5. Flow cytometry dot plots showing Annexin V^cy3^/PI cytotoxicity results for empty cationic liposomes in A549 cells. Result shows dot plots of A549 cells after 4 hrs incubation with empty liposomes at a total lipid concentration of (b) 1.0, (c) 5.0, (d) 10.0, (e) 25.0 and (f) 50.0 mM and a constant ratio of DC-Chol/DOPE, 1:1. A549 cells without liposomes were used as negative controls (a). The cells assessed using a Beckman Coulter MoFlo (minimum 10,000 cells/sample), and data analysed using Weasel software.

******

**Supplementary Figure S6. Dose-response profiles showing LDH release from A549 cells on exposure to empty liposomes.** Data from the LDH assay; expressed as LDH release and presented as the mean ± SD (N=3, n=6). Different concentrations of cationic liposomes (1.0, 5.0, 10.0, 25.0, and 50.0 mM) were used at different ratios of a DC-Chol:DOPE and incubated with cells for 4 hrs: a) 0.33:1, b) 0.5:1, c) 0.66:1, d) 1:1, e) 1.5:1, f) 2:1 and g) 3:1. Dose-response curves were generated using GraphPad Prism (v6).

**Supplementary Figure S7. Relative luciferase activity of A549-Luc cells after incubation with luc-siRNA-liposomes.** Cells were seeded onto 24-well plates at a density of 5 × 10^4^ cells per well and cultured overnight. Liposome-siRNA formulations were prepared at an N/P ratio of 3.125:1 and different DC-Chol:DOPE molar ratios, and applied to the cells for 4 hrs. A549-Luc cells were also treated with scrambled siRNA-liposomes (1:1 molar ratio of DC-Chol:DOPE, N/P ratio of 3.125:1 and applied at 1 μg siRNA/well) as non-targeting siRNA control, and the transfection reagent Lipofectamine® RNAiMAX siRNA delivery was used as positive control. A549-Luc cells not treated with Luc-siRNA liposomes taken as 100% of luciferase protein activity and used to normalise the luciferase activity of all tested formulations. Samples were removed and replaced with fresh medium, and the cells then incubated for a further 48 hrs before analysis. **** and *** indicate a significant difference between the results (p<0.0001 and p<0.001, respectively) and ns indicates no significant difference (p>0.05). Data is shown as the mean ± SD, (N=2, n=4).

**Supplementary Figure S8. Relative luciferase activity of A549-Luc cells after incubation with 1 μg/well of Luc-siRNA.** Cells were seeded onto 24-well plates at a density of 5 × 10^4^ cells per well and cultured overnight. Liposome-siRNA formulations were prepared at an N/P ratio of 3.125:1 and different DC-Chol:DOPE molar ratios and applied to the cells for 4 hrs. A549-Luc cells were also treated with the transfection reagent Lipofectamine^®^ RNAiMAX as positive control. A549-Luc cells not incubated with siRNA-Luc liposomes were used as 100% luciferase activity. Samples were removed and replaced with fresh medium, and cells were then incubated for a further 24, 48, 72 and 96 hrs before analysis. * and ** indicate a significant difference between the results (p<0.05 and p<0.01, respectively) and ns indicates no significant difference (p>0.05). Data is shown as the mean ± SD, (N=2, n=4).

**Supplementary Figure S9. Dose-response curves showing relative percentage cell viability of the endocytosis inhibitors after incubation for 4 hrs with A549 cell line.** Data are the results of an MTS assay and are expressed as relative cell viability and presented as the mean ± SD (N=3, n=6). Dose-response curves were generated using GraphPad Prism (v6).

**Supplementary Figure S10. Effect of endocytosis inhibitors on the uptake of Tƒ and CTß in A549 cells.** Cells were pre-treated with three different concentrations of inhibitors for 30 min, followed by 4 hrs of exposure to Alexa fluor 488 Tƒ, a clathrin-mediated pathway marker and Alexa fluor 488 CTß, a caveolin-mediated pathway marker. Uptake was assessed using a Beckman Coulter MoFlo (minimum 10,000 cells/sample). ****, ***, ** and * indicate statistical differences at p<0.05, p<0.01, p<0.001 and p< 0.0001, respectively, compared to the control (cells incubated with markers in absence of inhibitors), whereas ns indicates a non-significant result, p>0.05 (N=2, n=4).

***
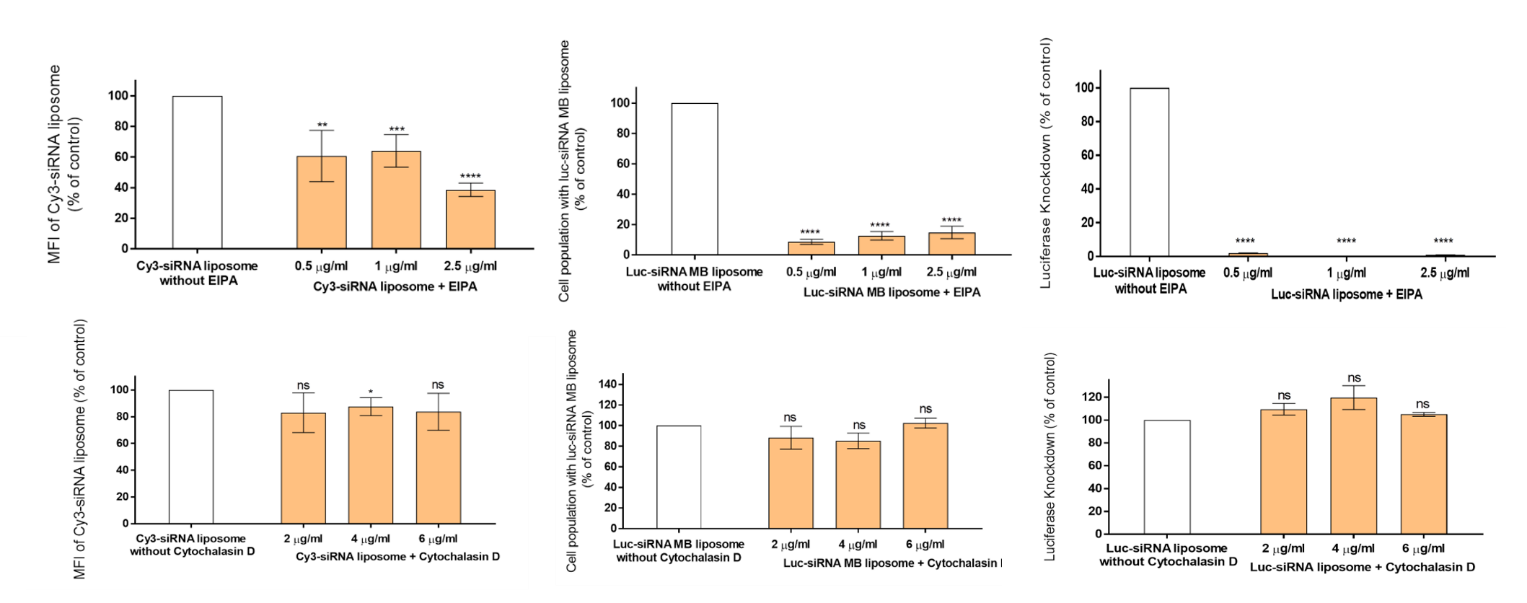
***

**Supplementary Figure S11. Effect of different sub-EC_50_ concentrations of EIPA and cytochalasin D inhibitors on internalization of siRNA complexes, engagement of liposomes delivered luc-siRNA-molecular beacon (MB) with target mRNA and silencing in A549-luc cells. Left:** Cells were pre-treated with three different concentrations of inhibitors for 30 min, followed by 4 hrs of exposure to Cy3-siRNA-liposomes. Uptake was assessed using a Beckman Coulter MoFlo (minimum 10,000 cells/sample). ****, ***, ** and * indicate statistical differences at p<0.05, p<0.01, p<0.001 and p< 0.0001, respectively, compared to the control (cells incubated with markers in absence of inhibitors), whereas ns indicates a non-significant result, p>0.05 (N=2, n=4). **Middle**: Fluorescence from flow cytometry experiments expressed relative to the control (cells without inhibitors representing 100%); data represent the mean ± SD (N=2, n=4), **** indicate a significant difference between the results (p<0.0001) and *ns* indicates the difference is a non-statistically significant (p>0.05) compared to the control. **Right:** *Luc*-siRNA-liposomes prepared at an N/P ratio 3.125:1, DC-Chol:DOPE ratio of 1:1, applied at 1 μg of *luc*-siRNA *per* well and 1 mM total lipid content. The *luc*iferase activity assessed after 48 hours. Luciferase knockdown relative to the control (cells without inhibitors as 100%), data represents the mean ± SD (N=2, n=4), **** indicate a significant difference between the results (p<0.0001) and *ns* indicates the difference is a non-statistically significant (p>0.05) compared to the control.

***Table S1: Selected concentrations of applied inhibitors and corresponding cell viability.*** Based on the results of the MTS screening (Fig. S9) and the uptake of pathways markers (Fig. S10), concentrations of the inhibitors were selected for use in investigating the mechanism of endocytosis of siRNA-liposomes. These concentrations show a low cytotoxic effect, as summarised in the table, while significantly inhibiting internalisation of specific ligands.

| Inhibitor | Concentration  (μg/ml) | Cell viability (%)  A549 A549-Luc | |
| --- | --- | --- | --- |
| Concanavalin A | 100 | 98.2 | 100 |
| Chlorpromazine | 20 | 80.4 | 88.9 |
| Dynasore | 20 | 89.5 | 96.1 |
| Genistein | 15 | 86.4 | 79.3 |
| Filipin | 20 | 80.9 | 97.5 |
| MβCD | 300 | 91.7 | 96.1 |
| Nystatin | 20 | 84.9 | 82.9 |
| EIPA | 0.5, 1.0, 2.5 | 90.8, 84.7, 79.3 | 95.2, 88.7, 84.3 |
| Cytochalasin D | 2, 4, 6 | 112.8, 95.6, 95.8 | 95.1, 89.6, 90.7 |


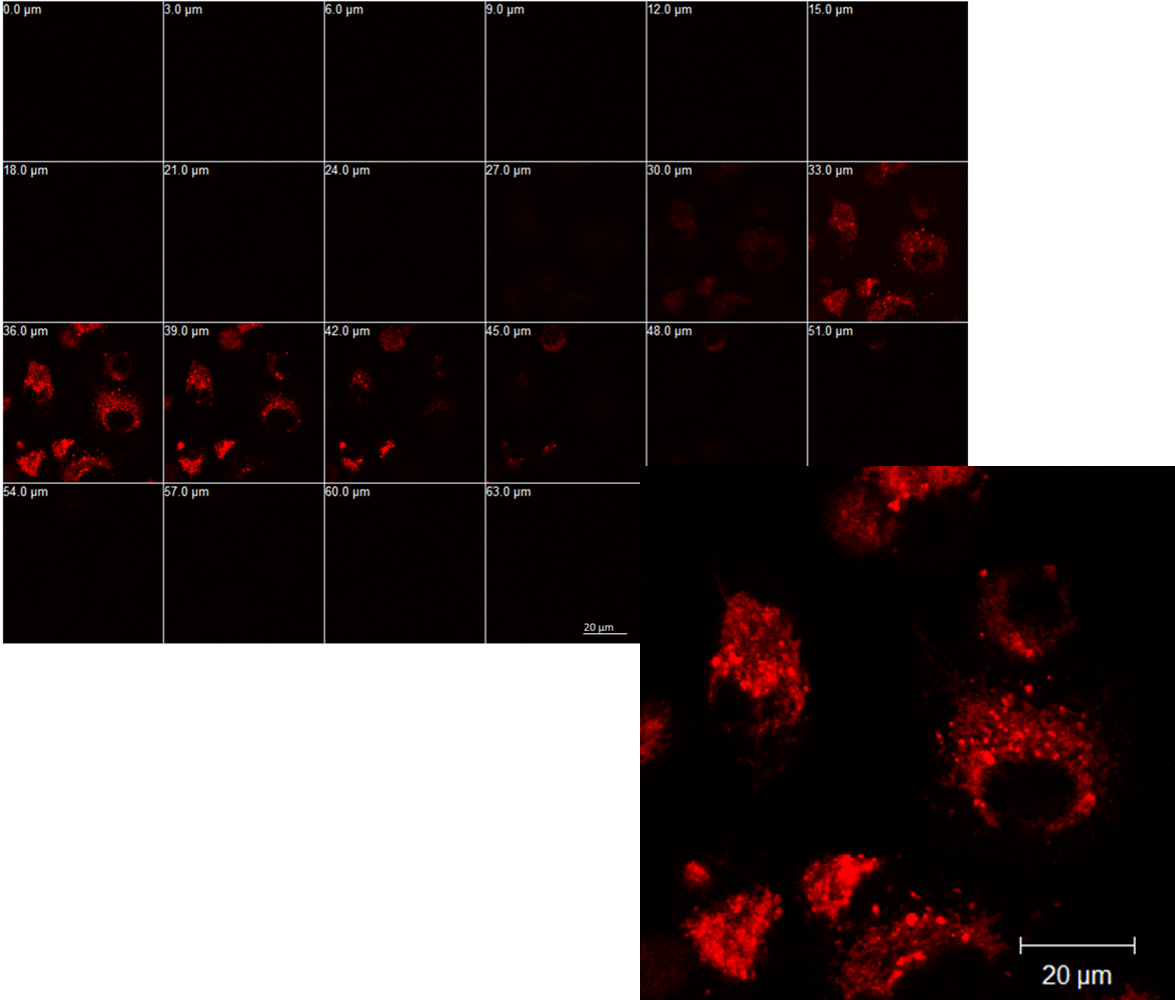


**Supplementary Figure S12. Confocal microscopy panel of z-stack micrographs of ^cy3^siRNA-liposomes cellular uptake in A549 cells**. Cells were incubated with cy3-siRNA-liposomes at an N/P ratio of 3.125:1 and a DC-Chol:DOPE ratio of 1:1 for 4 hrs and then assessed using confocal microscopy. Z-stack sections images from the top to the bottom of the cells are presented in individual panels at depths as illustrated. Scale bar: 20 μm. Zeiss LSM510 confocal microscope was used to view the fluorescence.


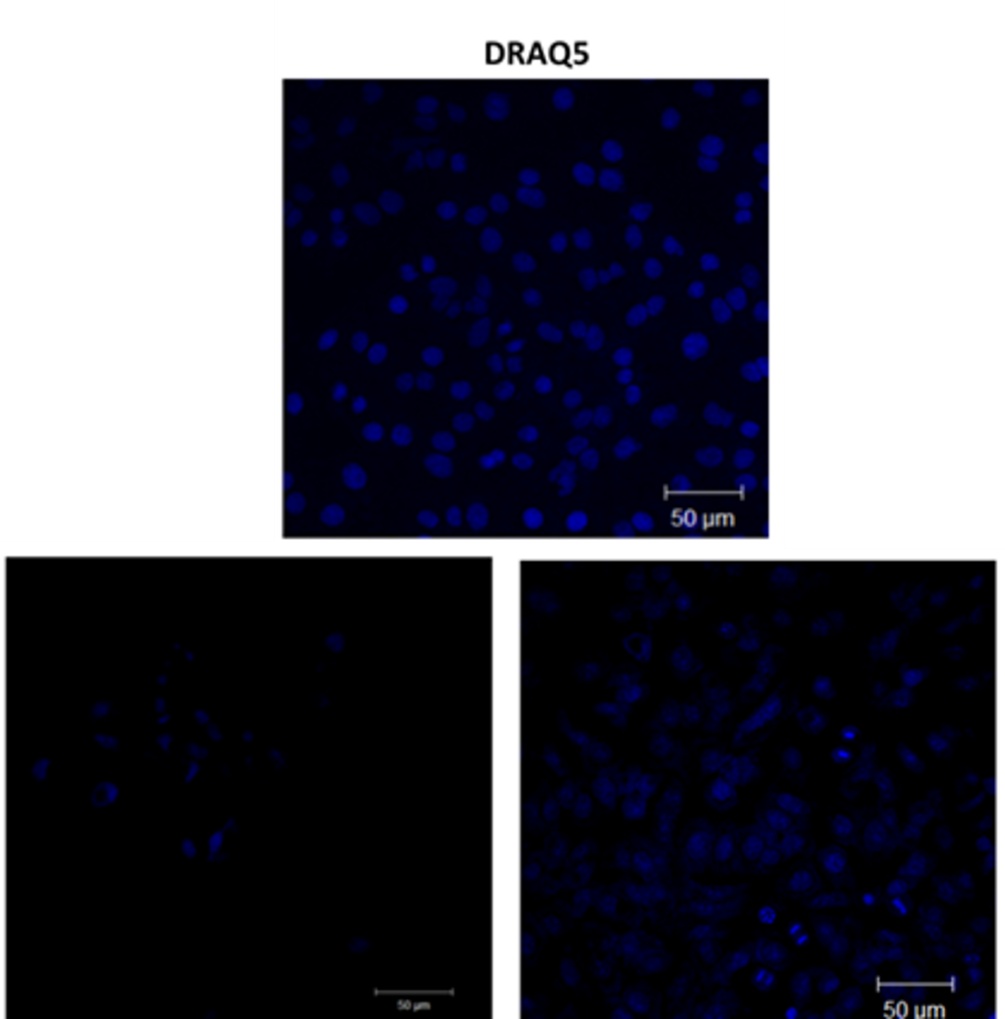


**Supplementary Figure S13. The effect of cytochalasin D inhibitor on the morphology of cell nuclei in A549 cells.** Confocal microscopy micrographs taken of A549 cells taken in the absence (upper micrograph) and presence (lower micrographs) of cytochalasin D (2 μg/ml). Nuclei appear blue as stained with DRAQ5. Zeiss LSM510 confocal microscope used to view the fluorescence.
